# Supplementary figures and images for: Community shelter use in response to two benthic decapod predators in the Long Island Sound
Source: PeerJ. 2016 Jul 27;4:e2265. doi: 10.7717/peerj.2265 (PMC4975000; doi:10.7717/peerj.2265)

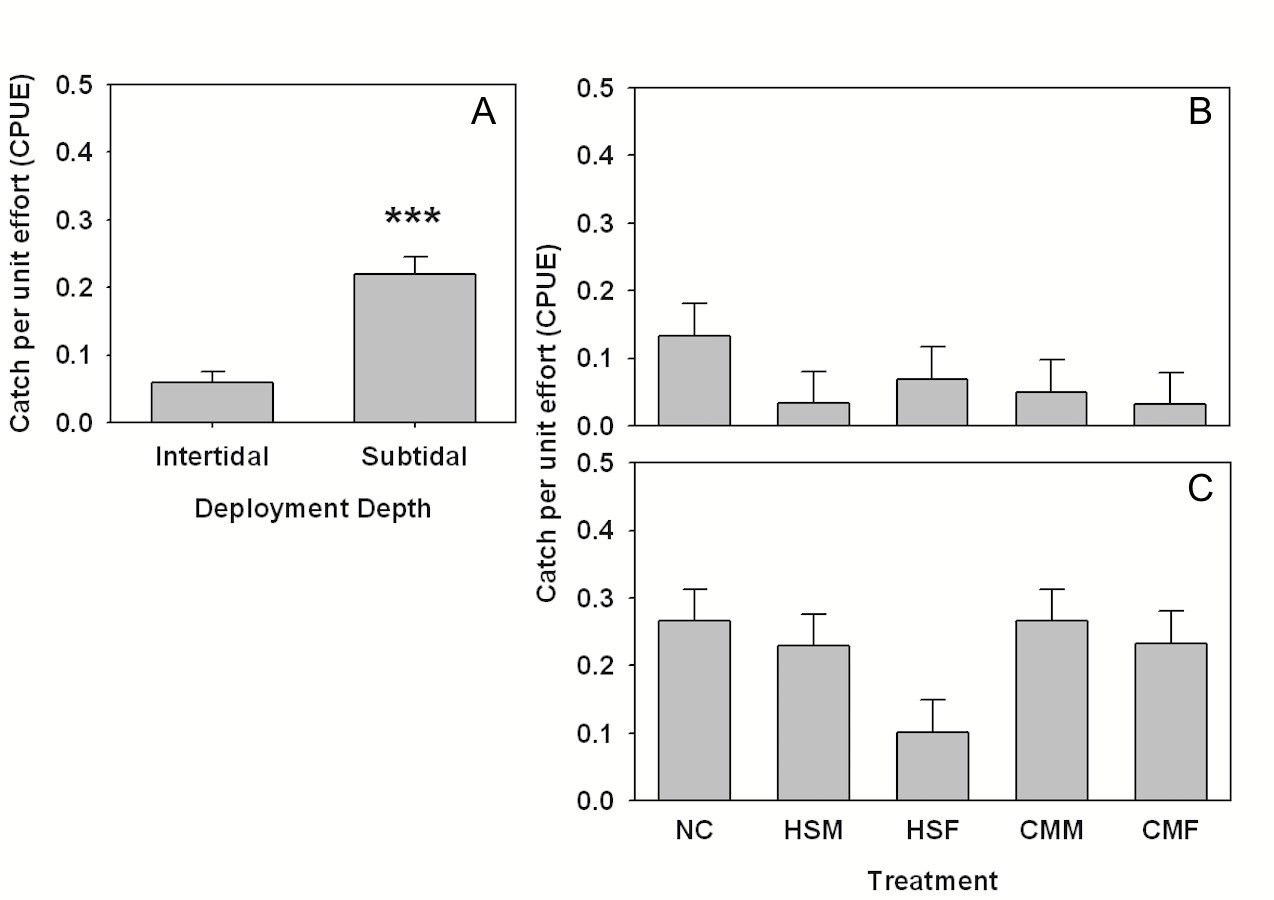

Supplement: Supplemental Information 2 — Catch per unit effort (CPUE, in number of animals caught per number of tubes (n = 60 tubes for each bar)) mean ± SE. (A) C. maenas use of subtidally- and intertidally-deployed shelters. (B) Summer intertidal deployment of multiple treatments (Treatments: NC = No Crab, HSM = Hemigrapsus sanguineus Male, HSF = H. sanguineus Female, CMM = C. maenas Male, and CMF = C. maenas Female). (C) Summer subtidal deployment in C. maenas. Significance between treatments or deployment depth indicated by: “*” = p < 0.05, “**” = p < 0.01, “***” = p < 0.001. [file peerj-04-2265-s002.png]

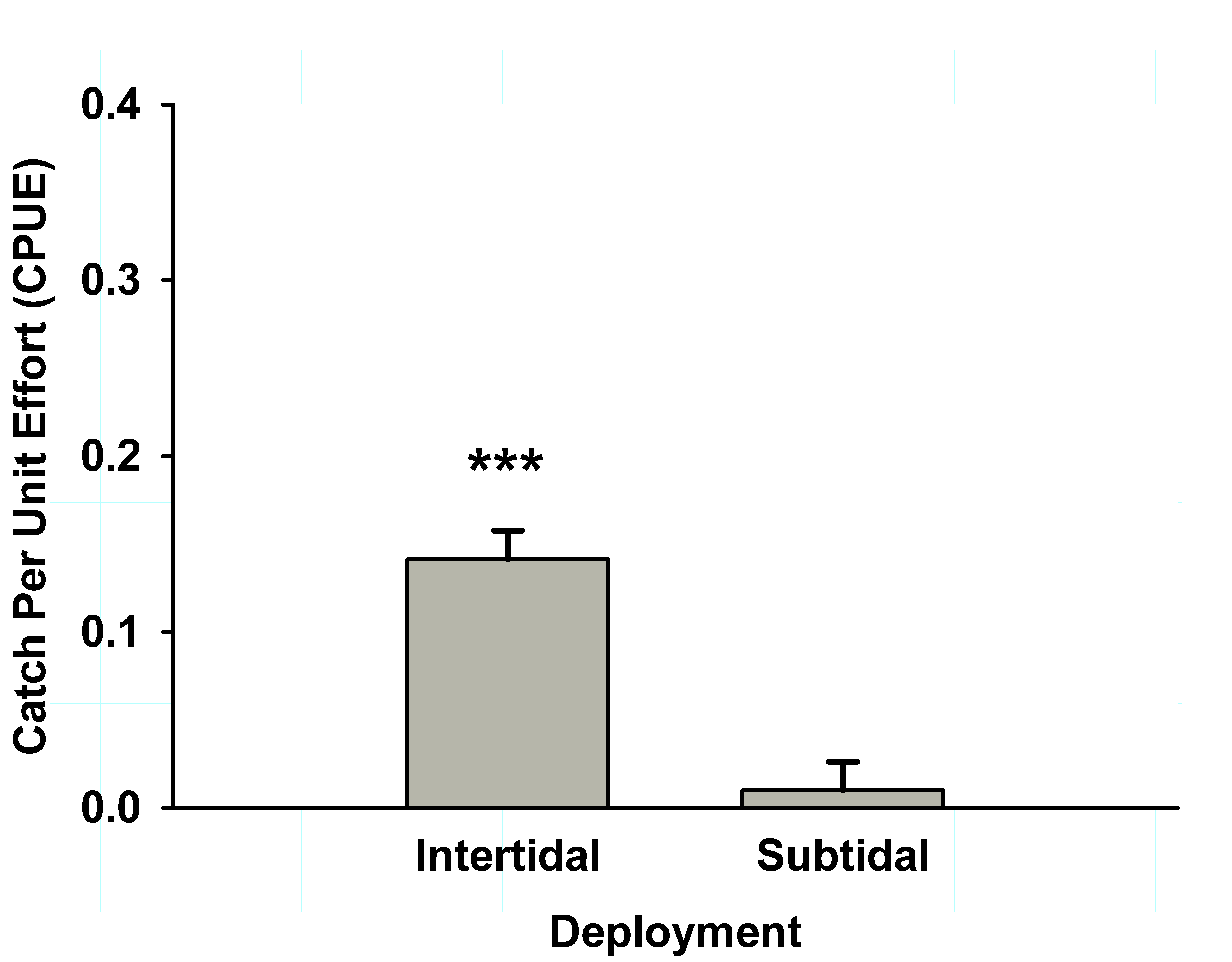

Supplement: Supplemental Information 3 — Stated in catch per unit effort (CPUE, in number of animals caught per number of tubes, n = 300 tubes for each bar) mean ± SE. Significance between deployment depth indicated by: “*” = p < 0.05, “**” = p < 0.01, “***” = p < 0.001. [file peerj-04-2265-s003.png]
